# Differential Size Distribution and Estrogen Receptor Cargo of Oviductal Extracellular Vesicles at Various Stages of Estrous Cycle in Mice

**DOI:** 10.1007/s43032-022-00862-w

**Published:** 2022-02-08

**Authors:** Chenchen Yi, Ya Ni, Peibei Sun, Tian Gao, Kun Li

**Affiliations:** Institute for Reproductive Health, Hang Medical College, Hangzhou, 310013 Zhejiang China

**Keywords:** Extracellular vesicles, Exosomes, Oviduct, Oviductal extracellular vesicles, Estrogen receptor, Vesicles size, Estrus

## Abstract

Oviductal extracellular vesicles (OEVs) play an important role in fertilization and embryo development. However, it remains largely unknown whether the size and protein cargo of OEVs change during the estrous cycle in mice. This study analyzed the changes in the size distribution and protein cargo of OEVs at four stages of the estrous cycle in mice. The distribution widths of OEVs according to the estrous cycle stage were as follows: proestrus, 20–690 nm in diameter, with two peaks at 50 and 250 nm; estrus, 22–420 nm in diameter, with two peaks at 40 and 200 nm; metestrus, 30–70 nm diameter, with a single peak at 40 nm; and diestrus, 10–26 nm diameter, with a single peak at 20 nm. The estrogen receptor (ER) level in OEVs at the proestrus stage differed significantly from that at estrus (*P* = 0.013) and diestrus (*P* = 0.005). The levels of CD9 and Hsc70 fluctuated across the four stages, although with no significant differences. Furthermore, OEVs were observed among the cilia and microvilli of epithelial cells at the proestrus, estrus, and diestrus stages, but not at the metestrus stage. The number of observed OEVs was the highest at the proestrus stage, followed by the estrus, and the diestrus stage. Endosomes were also observed at the estrus and diestrus stages. The change of the OEV size and ER cargo is associated with the estrous cycle in mice. Our findings increase the understanding of the physiological characteristics of OEVs, which may have clinical applications.

## Introduction

Oviductal extracellular vesicles (OEVs) that are released from the oviduct play important roles in the development of sperm, oocytes, and embryos [1–3]. OEVs have been increasingly documented to have the capacity to advance sperm function and fertilizing ability [4, 5], improve egg maturation and blastocyst yield, and enhance embryo development and quality, via transferring their cargo [6–9]. OEVs carry various biological cargoes, including proteins, mRNA, miRNA, and lipids, and are incorporated into the recipient cells and regulate their development capacity or improve their function; thus, OEVs have potential clinical application as molecular transmitters or therapeutic vectors [10, 11].

The size distribution and cargoes of OEVs may affect the function and application of exosomes OEVs, for the size of extracellular vesicles is a crucial determinant of their incorporation into recipient cells [12]. The differences in size distribution may be related to the different animal models or methods used [13]. Protein cargoes of OEVs in different species have been revealed [14, 15] and OEVs in mice carry various protein markers, including CD9 and Hsc70[16]. However, across the different stages of the estrous cycle in mice, whether the size and protein cargo of OEVs change remain largely unknown. In mammals, the estrous cycle refers to cyclic physiological changes under the action of sex hormones secreted after sexual maturity. In mice, each estrous cycle is divided into four stages: the proestrus, estrus, metestrus, and diestrus stages [17]. The estrous cycle is regulated by endocrine hormones, including prolactin (PRL), luteinizing hormone (LH), follicle-stimulating hormone (FSH), progesterone (P4), and estradiol (E2) [18]. The function and biological roles of the oviduct are regulated by these hormones during the estrous cycle [19, 20]. Thus, this study aims to provide the characteristics of OEVs for the potential clinical application, based on the hypothesis that the characteristics of OEVs change in mice across the different stages of the estrous cycle.

In this study, we investigated the changes in OEVs at the proestrus, estrus, metestrus, and diestrus stages in mice. We examined the OEV size distribution; changes in the protein cargo carried by OEVs comprising estrogen receptor (ER), CD9, and Hsc70; and the ultrastructure of the OEV-releasing oviduct. The specific components of the OEV protein cargo were selected; the ER is dynamically expressed in the female reproductive tract during the estrous cycle [21]; CD9 is one of the most abundant proteins on the exosomal membrane [16, 22, 23]; and Hsc70 is one of the highly conserved members of the heat shock protein 70 (Hsp70) family; Hsp70 is a known exosomal protein marker and positive control reported in exosome proteomic studies [24, 25]. This study elucidated the characteristics and dynamics of OEVs at different stages of the estrous cycle and thus may be useful for revealing physiological and pathological changes associated with the estrous cycle. It also provides new insights on how to obtain OEVs or extracellular vesicles with uniform size and stable protein cargoes from other sources, which may have clinical applications [10, 11, 13].

## Materials and Methods

### Reagents and Materials

Rabbit monoclonal anti-CD9 antibody (EPR2949, ab92726; Abcam, Shanghai, China), rabbit monoclonal anti-Hsc70 antibody (EP1531Y, ab51052; Abcam, Shanghai, China), and rabbit monoclonal anti-ER alpha antibody (E115, ab32063; Abcam, Shanghai, Chin*a*) were used in this study. Normal rabbit IgG was obtained from R&D Systems (Minneapolis, MN, USA). Filtered and sterile phosphate-buffered saline (PBS) were purchased from Sigma-Aldrich Corporation (St. Louis, MO, USA). A pre-stained protein ladder was obtained from Thermo Fisher Scientific (Baltics UAB, Vilnius, Lithuania).

### Animals

Female ICR mice aged > 8 weeks (20–25 g) were purchased from the Experimental Animal Center of Zhejiang Province (License number: SCXK (Zhe) 2019–0002) and Shanghai Jihui Experiment Animal Feeding Limited Co. (License number: SCXK (Hu) 2017–2012). The animals were housed in plastic cages under specific pathogen-free conditions in a temperature-controlled room (23 ± 2 °C) with 60% ± 10% relative humidity and a 12 h light–dark cycle. The study protocol was reviewed and approved by the Animal Ethics Committee of Zhejiang Academy of Medical Sciences. At least 78 mice, ranging from 12 to 34 for different groups, were used for each experiment; the experiments were repeated four times; and all 334 mice were used to obtain sufficient OEVs for further experiments given the small size of the mouse oviduct.

### *Evaluation of Different Stages of the Estrous Cycle *via* Vaginal Cytology*

**Fig. 1:**
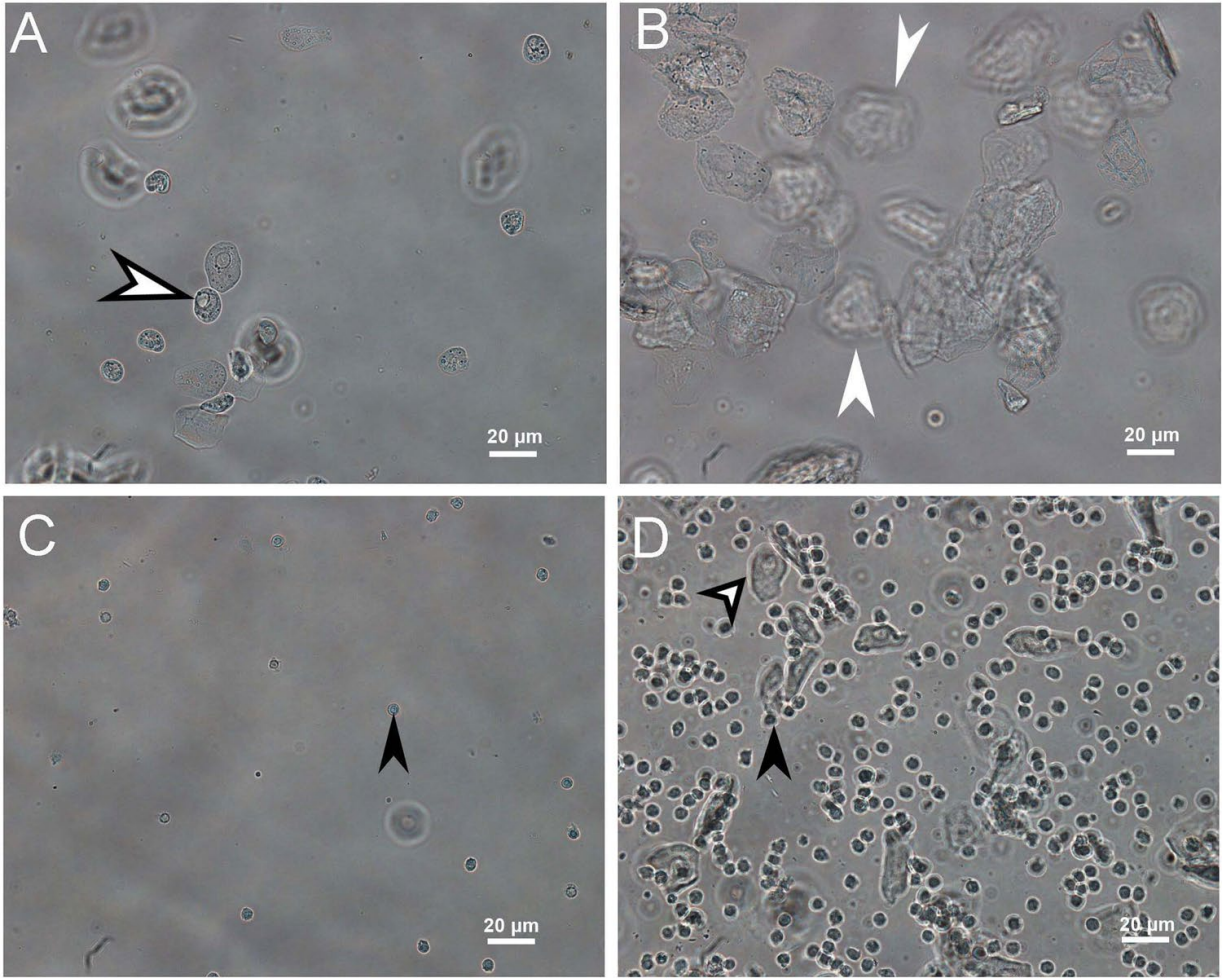
Cell morphological evaluation of vaginal secretions at different stages of the estrous cycle. (A) Proestrus stage: mainly epithelial cells with a round nucleus (white arrow with a black edge). (B) Estrus stage: cornified squamous epithelial cells with dense clusters (white arrow). (C) Metestrus stage: predominantly small leukocytes (black arrow). (D) Diestrus stage: rarely squamous epithelial cells with fragments (black arrow) and epithelial cells with a round nucleus (white arrow with a black edge) but predominantly small leukocytes. The cells were observed under a microscope (Eclipse 80i; Nikon Inc., Tokyo, Japan) with 200 × magnification. Scale bar = 20 μm

To identify the estrous cycle stage in each mouse, vaginal cells were collected through vaginal lavage. Sterile PBS (50 µL) was injected into the vaginal canal and aspirated using a pipette, and the process was repeated 10 times. The aspirated PBS containing vaginal cells were collected into a clean Eppendorf tube, and 10 μL of it was placed on a glass slide and covered with a 22 mm × 22 mm coverslip. The cells were observed under a microscope (Eclipse 80i; Nikon Inc., Tokyo, Japan) at 200 × magnification. Each mouse was classified into one of the four stages (proestrus, estrus, metestrus, and diestrus) as reported [, 17, 18], based on the types and numbers of vaginal cells observed under the microscope. The cell types were classified immediately after sample collection to identify the stage of the estrous cycle. When only epithelial cells with a round nucleus (white arrow with a black edge) were observed, the stage was judged as the proestrus stage (Fig. 1A); when cornified squamous epithelial cells with dense clusters (white arrow) were observed, the estrus stage (Fig. 1B) was identified; when predominantly small leukocytes (black arrow) were seen, the metestrus stage (Fig. 1C) was identified; and when predominantly continuous leukocytes (black arrow), and rarely squamous epithelial cells with fragments and squamous epithelial cells with a round nucleus (white arrow with a black edge), were observed, the stage was judged as the diestrus stage (Fig. 1D). Each mouse was classified into a specific estrous cycle stage for further experiments.

### OEV Extraction and Preparation

After classification into the different stages of the estrous cycle, the mice were euthanized by CO_2_ overdose. After mincing the oviducts, luminal fluids were collected in sterile and filtered PBS (Sigma) with or without protease inhibitors, as described by Bathala et al. [5]. Clarified luminal fluids were centrifuged at 3500 × *g* for 10 min to pellet blood cells and excess tissue debris, and the supernatant was further centrifuged at 14,000 × *g* for 30 min at 4 °C to remove the remaining debris. The supernatant in 10.4-mL polycarbonate bottle with cap (catalog# 355603, Beckman) was balanced using sterile and filtered PBS (Sigma) and ultracentrifuged at 120,000 × *g* for 2 h at 4 °C using the Beckman Coulter Optima EX-90 ultracentrifuge and the Type 70 Ti/70.1 Ti (k-factor 44) rotor. The supernatant was discarded, and the pellets were resuspended in PBS containing a protease inhibitor (Complete Mini EDTA-free Protease Inhibitor Cocktail; Roche Diagnostics Deutschland GmbH, Mannheim, Germany). The protein concentration of the OEV pellet suspension was measured using a bicinchoninic acid (BCA) kit (Beyotime Institute of Biotechnology, Shanghai, China), and the samples were stored at – 80 °C until use.

### Measurement of OEV Size Distribution

The OEV size distribution was measured using dynamic light scattering on a Zetasizer Nano ZS (ZEN3600) instrument (Malvern Instruments, Malvern, UK), as described by Serrano-Pertierra et al. [26]. The intensity of scattered light at 173° was measured using a solid-state He–Ne laser at 633 nm. All measurements were performed in triplicate at 25 °C. The samples were diluted with PBS (pH 7.4) as required. Data processing and analysis were performed using the Zetasizer software, and the results were acquired automatically.

### OEV Staining for Transmission Electron Microscopy (TEM)

OEV staining for TEM was performed according to a method described by Bathala et al. [5]. TEM copper grids (size 200 mesh) with a formvar/carbon film were floated in the OEV suspension, washed with water, and stained with 2% phosphate tungsten before TEM (HT7700; Hitachi, Tokyo, Japan) imaging and analysis.

### Sodium Dodecyl Sulfate–Polyacrylamide Gel Electrophoresis (SDS-PAGE) and Western Blotting

SDS-PAGE and Western blot analysis were performed as described by Li et al. [27]. The protein concentration of OEVs was measured using a BCA protein assay kit from Beyotime Institute of Biotechnology (Shanghai, China). Equivalent amounts of protein were resolved by SDS-PAGE with 10% acrylamide gels and electrotransferred to Immunoblot-P membranes (Millipore Corporation, Bedford, MA, USA). The membranes were incubated overnight with the anti-CD9, anti-ER, or anti-Hsc70 primary antibodies at 4 °C. Subsequently, the membranes were washed three times (5 min each) using TBS (Tris-buffered saline) containing 0.01% (v/v) Tween-20 and incubated with TBS-blocking solution (pH 7.4) containing 5% non-fat milk for 2 h at room temperature. Then, the membranes were incubated with a peroxidase-conjugated secondary antibody (1:5000) at room temperature for 1 h, followed by washing three times with TBST. Protein bands on the membranes were probed using a SuperSignal™ West Femto Maximum Sensitivity Substrate kit (Thermo Scientific, Rockford, IL, USA) following the manufacturer’s instructions. The molecular weights of the detected target proteins were inferred using the pre-stained protein markers. The target proteins were quantified via the gray intensity of their bands analyzed by ImageJ software and the amount of loaded protein if needed.

### Immunoelectron Microscopy

The immunogold-labeling was performed according to the method described by Bathala et al. [5]. OEVs were mixed with 4% paraformaldehyde (1:1) for 20 min before being placed on 200-mesh formvar/carbon copper grids. The grids were washed twice with PBS for 3 min each and then washed three times for 3 min with 100 μL PBS containing 50 mM glycine. The grids were blocked with 100 μL of 5% bovine serum albumin (BSA) blocking buffer for 30 min and then incubated with the primary antibodies, anti-CD9 (1:20), and anti-ER (1:20), at 4 °C overnight. The grids were washed with the buffer for 3 min (repeated six times) and incubated with the secondary antibody (1:20) labeled with colloidal gold for 30 min. Thereafter, the grids were washed with 5% BSA-blocking solution (repeated six times), further washed with PBS for 2 min (repeated six times), fixed with 1% glutaraldehyde solution for 2 min, and washed with deionized water for 2 min (repeated six times). The OEVs in the grids were negatively stained with uranyl acetate for 90 s before washing twice with deionized water for 2 min. After drying, the OEVs in the grids were examined with TEM (Tecnai G2 Spirit, Thermo Fisher Scientific) operated at 120 kV.

#### TEM

TEM was performed as described by Bathala et al. [5]. One oviduct tissue representative of each estrous cycle stage was fixed with filtered PBS containing 2% formaldehyde and 2% glutaraldehyde and stored at 4 °C. The tissues were treated with 1% osmium acid for 1 h, washed three times (10 min each) with water, and treated with 2% uranium acetate solution. Thereafter, the tissues were dehydrated using the graded series of ethanol (50%, 70%, 90%, 100% ethanol) and 100% acetone. The tissues were then treated with a mixture of embedding agent and acetone (1:1 at room temperature for 2 h, and 1:3 at room temperature for 3 h). Ultrathin sections of the tissues after polymerization were observed using a Tecnai G2 Spirit electron microscope (Thermo Fisher Scientific) operated at 120 kV.

### Statistical Analysis

The relative protein levels are expressed as the mean ± standard error of the mean. First, the homogeneity of variances of the experimental data was tested; if the *P*-value was > 0.05, statistical analyses were performed using the least significant difference test, one-way analysis of variance in SPSS 20.0 (IBM, Armonk, NY, USA), or Dunnett’s T3 test was performed[27]; if data were non‐normally distributed, Mann–Whitney test was used. Statistical significance was set at *P* < 0.05.

## Results

### Size Distribution of OEVs Released During the Four Stages of the Estrous Cycle

**Fig. 2:**
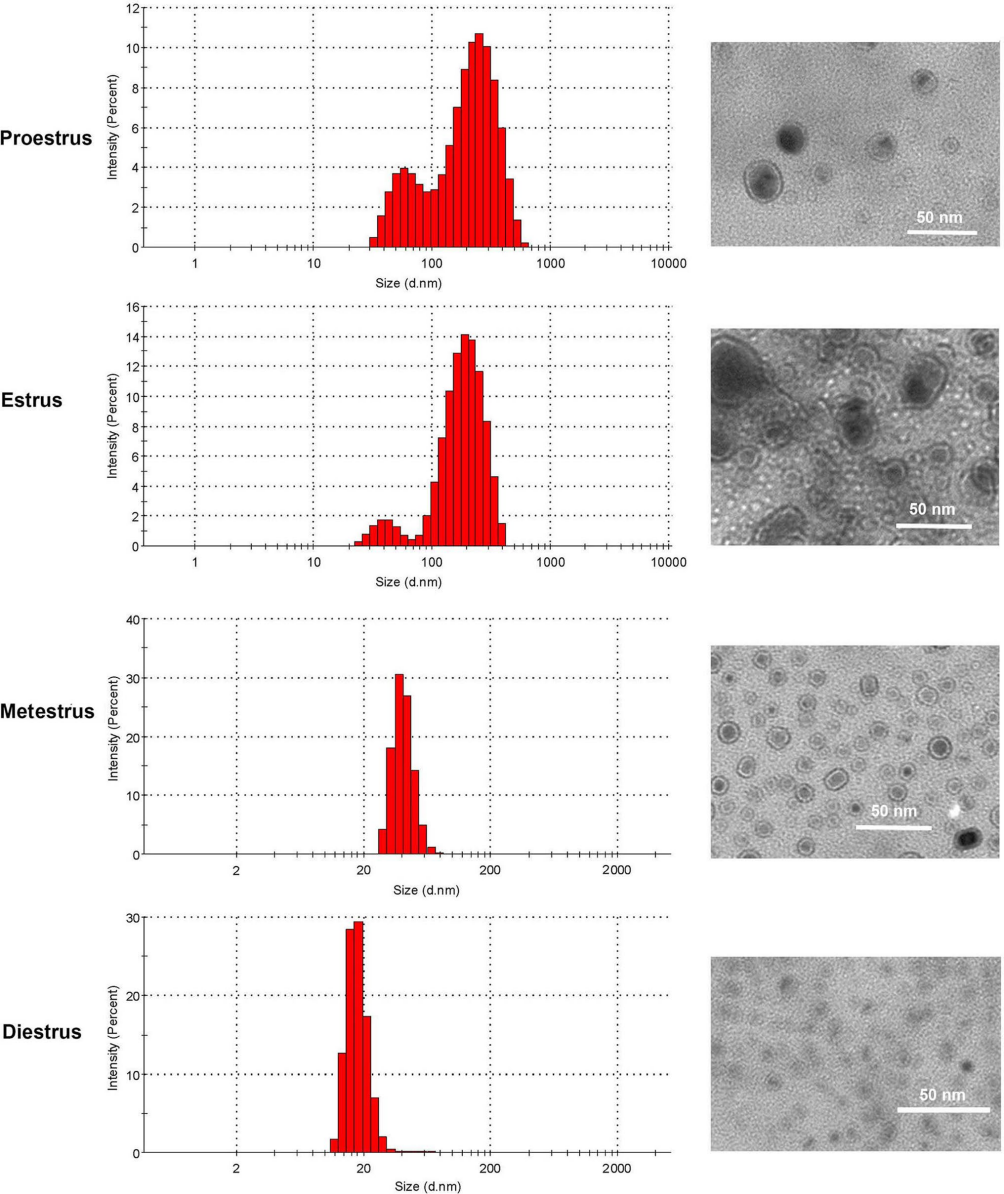
OEV size characteristics at different stages of the estrous cycle in mice. Left, the size distribution of OEVs; Proestrus OEVs (pooled from 14 mice), Estrus OEVs (pooled from 14 mice), Metestrus OEVs (pooled from 16 mice); Diestrus OEVs (pooled from 34 mice). The graphs illustrating results were automatically acquired using the Zeta sizer software. Right, confirmation of particle size of the corresponding samples on the left via transmission electron microscopic observation. Scale bar = 50 nm

The OEV distribution widths were determined to explore the changes in OEV size across the four stages of the estrous cycle. The distribution widths of OEV size at the different stages were as follows: proestrus, 20–690 nm in diameter, with two peaks at 50 and 250 nm; estrus, 22–420 nm in diameter, with two peaks at 40 and 200 nm; metestrus, 30–70 nm diameter, with a single peak at 40 nm; and diestrus, 10–26 nm diameter, with a single peak at 20 nm (Fig. 2, left). TEM was performed to further validate the OEV size. Sphere-like particles were observed, and their size matched the range of distribution widths (Fig. 2, right), suggesting that the OEV size was stable and reliable.

### Changes in the Levels of Proteins Carried by OEVs Released at Four Stages of the Estrous Cycle

**Fig. 3:**
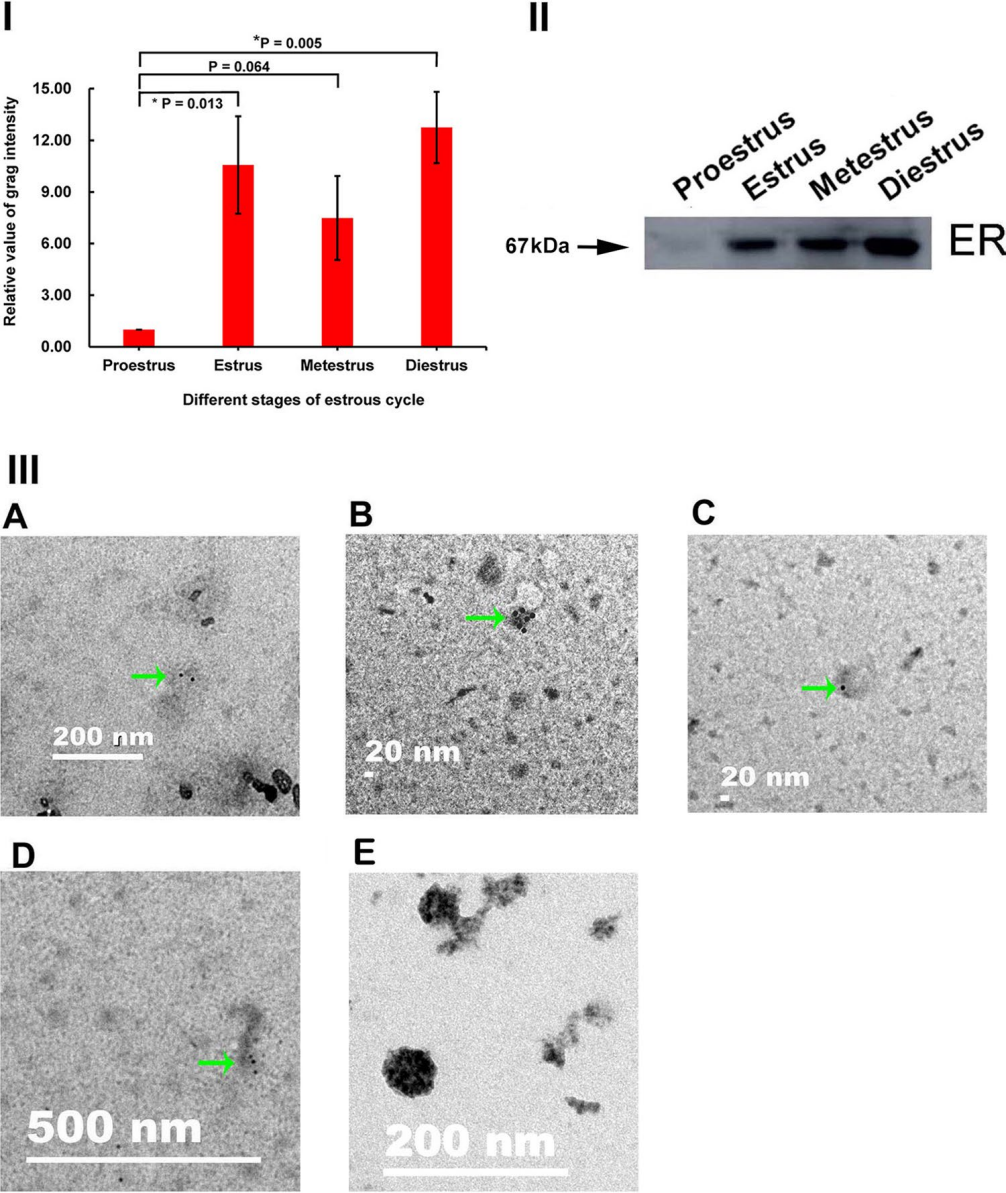
Quantitative analysis of ER in OEVs at different stages of the mouse estrous **cycle.** Protein from OEVs, 4.6 µg /lane, was loaded when Western blot was analyzed. (I) Quantitative analysis of ER levels in OEVs. The relative levels of ER are determined by standardizing each group with proestrus as 1.00. *P < 0.05, the ER level at the estrus (*P* = 0.013) and diestrus (*P* = 0.005) stages is statistically different from that at the proestrus stage. Data are presented as mean ± standard error of the mean, with the experiments repeated three times. (II) Western blot of ER in OEVs. (III) Validation of ER in OEVs using immunoelectron microscopy. (A) Proestrus, (B) estrus, (C) metestrus, (D) diestrus, (E) rabbit IgG control. The scale bar is shown as in the figure; the black gold particles, indicated by the green arrows, indicate that ER in the OEV was recognized by ER antibody

To explore the changes in OEV protein cargoes across the four stages of the estrous cycle, the representative OEV cargo proteins, i.e., ER, CD9, and Hsc70, were analyzed using Western blotting and immunoelectron microscopy. The ER is dynamically expressed in the female reproductive tract during the estrous cycle [21] and is a known protein marker. The level of ER in OEVs at the proestrus, estrus, metestrus, and diestrus stages in mice was detected by Western blotting (Fig. 3II). The highest ER level at the diestrus stage was approximately 13-fold higher than the lowest level at the proestrus stage, while the highest ER level at the estrus and metestrus stages was in between these two levels (Fig. 3I). Furthermore, the ER level at the proestrus stage differed significantly from that at the estrus (*P* = 0.013) and diestrus (*P* = 0.005) stages. The presence of ER was confirmed when black round colloidal gold particles were observed on immunoelectron microscopy, and no gold particles were observed when rabbit IgG was used as negative control (Fig. 3III). These results indicated that the ER levels in OEVs changed across the different stages of the estrous cycle.

**Fig. 4:**
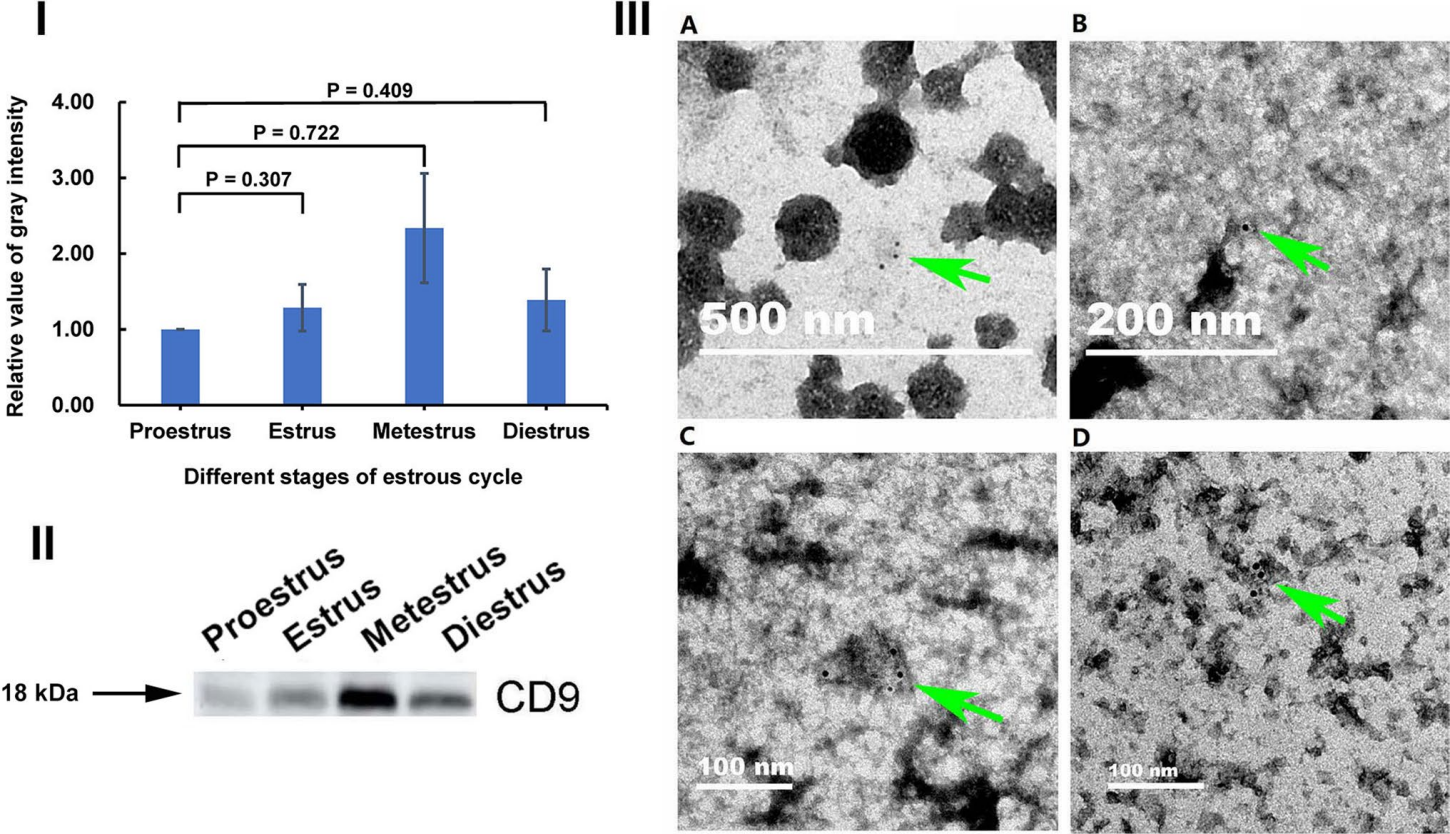
Quantitative analysis of CD9 in OEV at different stages of the mouse estrous cycle. Protein from OEVs, 4.8 µg /lane, was loaded when Western blot was analyzed. (I) Quantitative analysis of CD9 levels in OEV. The relative levels of CD9 are determined by standardizing each group with proestrus as 1.00. Data are presented as mean ± standard error of the mean, with the experiments repeated six times using three different samples. A P value less than 0.05 was statistically significant. (II)Western blot of CD9 in OEVs. (III) Validation of CD9 in OEVs using immunoelectron microscopy. (A) Proestrus, (B) estrus, (C) metestrus, (D) diestrus. The observation of dark gold particles indicates the presence of CD9. The scale bar is shown as in the figure; the black gold particles, indicated by the green arrows, suggest CD9 in the OEV were recognized by the CD9 antibody

**Fig. 5:**
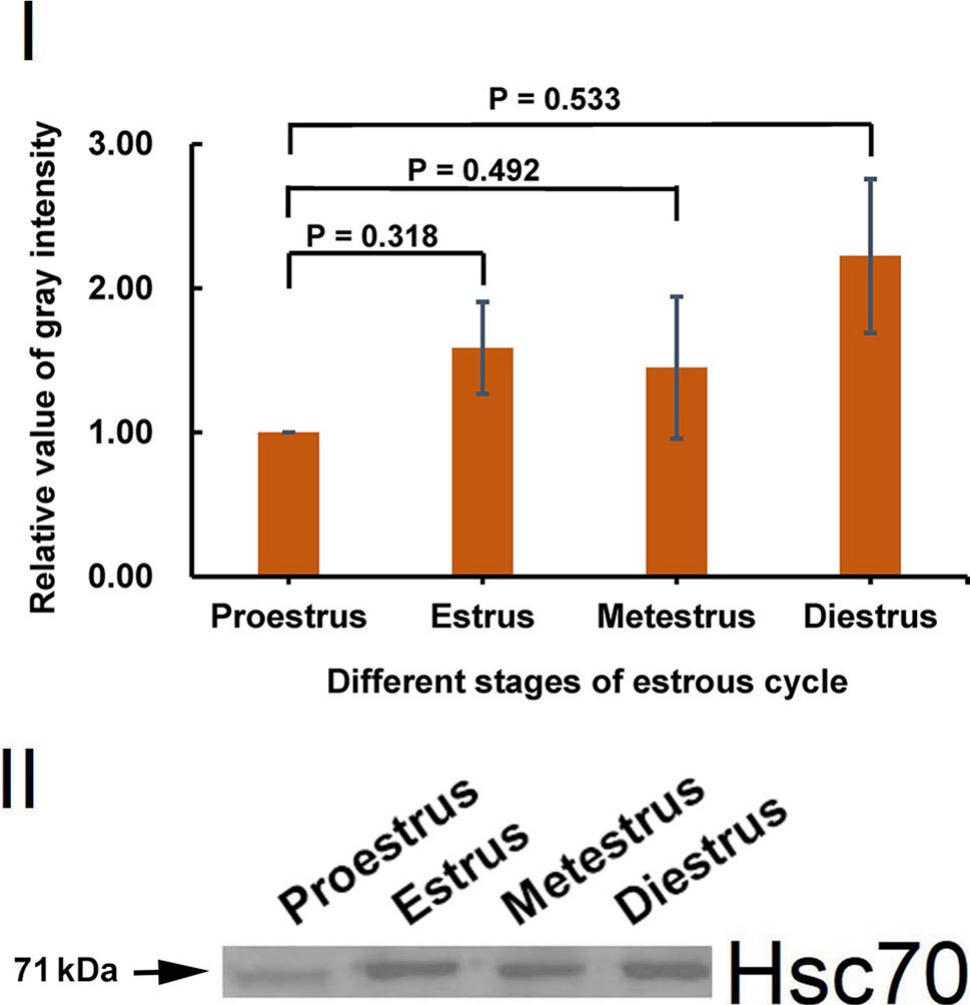
Quantitative analysis of Hsc70 in OEV at different stages of the mouse estrous cycle. Protein from OEVs, 7.5 µg/lane, was loaded when Western blot was analyzed. (I) Quantitative analysis of Hsc70 levels in OEVs. The levels of Hsc70 are determined by standardizing each group with proestrus as 1.00. Data are presented as mean ± standard error of the mean, with the experiments repeated six times using three different samples. A *P* value less than 0.05 was statistically significant. (II)Western blot of Hsc70 in OEVs

CD9 levels (Fig. 4II) and Hsc70 levels (Fig. 5II) in OEVs at four stages were detected by Western blotting. The levels of CD9 in OEVs fluctuated across the different stages, and the highest level at the metestrus stage was approximately 2.5-fold higher than the lowest level at the proestrus stage, whereas the CD9 levels at the estrus and diestrus stages were between these two levels (Fig. 4I); however, the difference in CD9 levels among the stages was not significant. The trend of change in the Hsc70 levels was different from that of CD9 but similar to that of the ER. The highest level at the diestrus stage was approximately twofold of the lowest level observed at the proestrus stage, and the level at the estrus and metestrus stages was approximately 1.5-fold higher than the lowest level at the proestrus stage (Fig. 5I); however, the difference among the stages was not significant. Moreover, black round colloidal gold particles observed on immunoelectron microscopy confirmed the presence of CD9 (Fig. 4III), and no gold particles were observed when rabbit IgG was used as negative control (Fig. 3III). These results indicated that the CD9 level and Hsc70 level fluctuate in OEVs across the different stages of the estrous cycle.

### Ultrastructure of the OEV-Releasing Oviduct at Four Stages of the Estrous Cycle

**Fig. 6:**
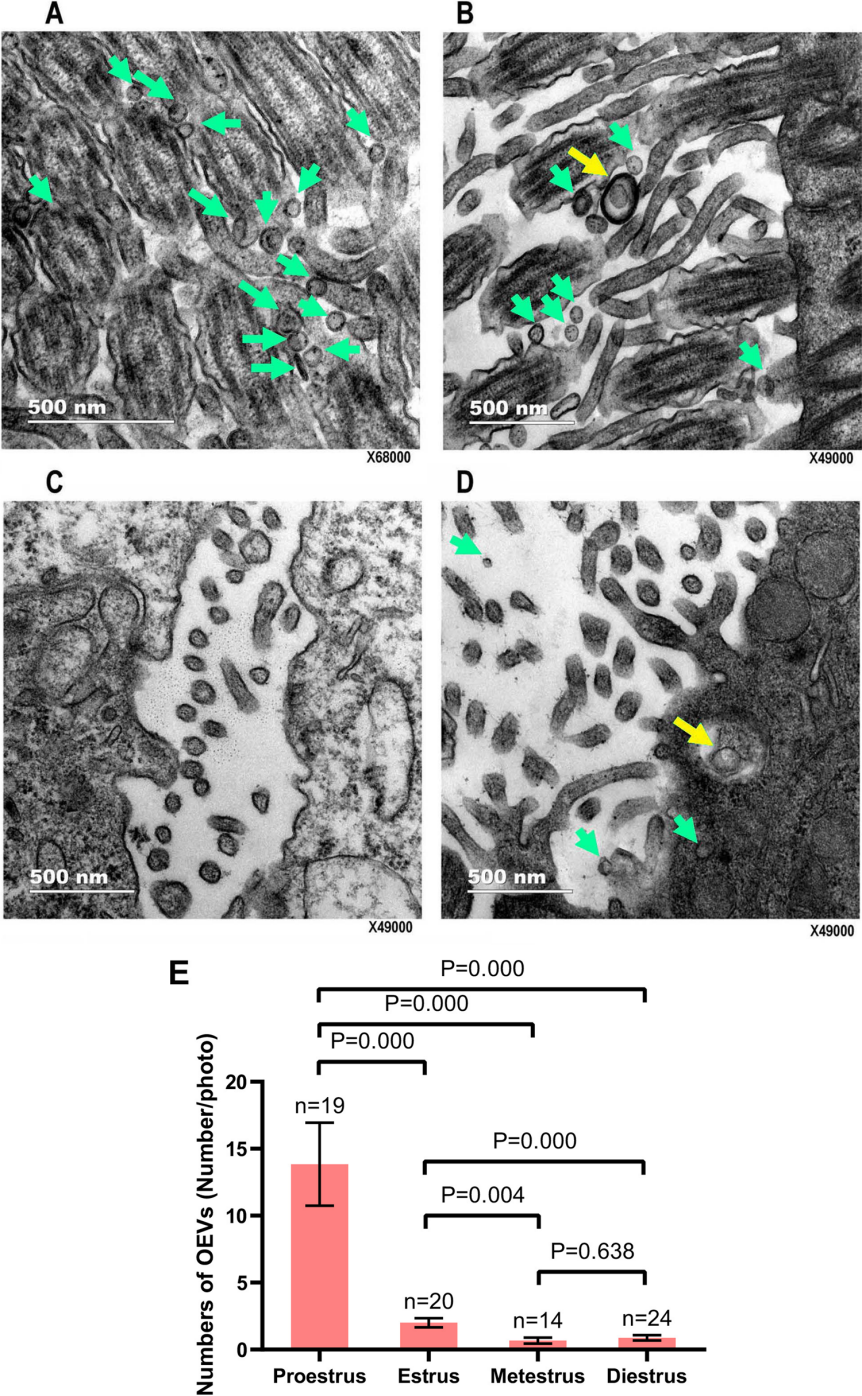
Ultrastructure of the oviduct and OEV secretion at different stages of the mouse estrous cycle. (A) Proestrus stage: thick cilia and microvilli are seen on the surface of the epithelial lining of the oviduct lumen; many OEVs (green arrows) are present among the thick cilia and microvilli, and the OEV sizes are different. Representative images from a total of 19 photographs. (B) Estrus stage: cilia and microvilli are seen on the surface of the epithelial lining of the oviduct lumen; OEVs (green arrows) are present among the thick cilia and microvilli, and endosomes (yellow arrow) are observed. Representative images from a total of 20 photographs. (C) Metestrus stage: only microvilli but no cilia are observed on the surface of the epithelial lining of the oviduct lumen. Representative images from a total of 14 photographs. (D) Diestrus stage: only microvilli but no cilia are observed. OEVs are hardly observed, but endosomes (yellow arrow) are seen. Representative images from a total of 24 photographs. (E) The number of OEVs in TEM photographs. Data are presented as mean ± standard error of the mean. n, the number of photographs; statistical analyses were performed using Mann–Whitney test. A *P* value less than 0.05 was statistically significant

Oviducts were subjected to TEM analysis to determine the oviduct ultrastructure at different stages of the estrous cycle and examine the status of the released OEVs. At the proestrus stage (Fig. 6A), thick cilia and microvilli were observed on the surface of the epithelial lining of the oviduct lumen, which contained several OEVs (green arrows) of different sizes. Moreover, the number of cilia was the highest, with more cilia than microvilli, at the proestrus stage compared to the other three stages. At the estrus stage (Fig. 6B), similar to the proestrus stage, cilia and microvilli were observed on the surface of the epithelial lining of the oviduct lumen, and OEVs (green arrows) were present among the thick cilia and microvilli. Endosomes (yellow arrow) were also observed in blebs. At the metestrus stage (Fig. 6C), only microvilli but no cilia were observed on the surface of the epithelial lining of the oviduct lumen. At the diestrus stage (Fig. 6D), only microvilli but no cilia were observed. OEVs were rarely observed, but endosomes (yellow arrow) were detected. The observed numbers of OEVs at the different stages are shown in Fig. 6E. The difference among the stages was statistically significant, except for metestrus vs. diestrus. These results indicated that the release of OEVs changes across the four stages of the estrous cycle.

## Discussion

To the best of our knowledge, this is the first study to provide evidence that in mice, the size distribution of OEVs changes across the different stages of the estrous cycle. We observed two peaks in the size distribution of OEVs at the proestrus and estrus stages, and only a single peak at the diestrus and metestrus stages. This pattern of OEV size distribution in mice is different from that observed in cattle [24]. In the previous study, the OEV size distribution was > 30 nm, and only one peak was observed in the range of 30–100 nm regardless of the stage of the estrous cycle [24]. In mice in the superovulated cycle, the size distribution of OEVs varies from < 100 nm in exosomes to 100–1000 nm in microvesicles [5]. This difference in size distribution in different studies can be attributed to differences in animal models and methods used, as previously reported [12]. Therefore, this study furthers our understanding of how cells produce different sizes of extracellular vesicles, especially when extracellular vesicles are applied [13].

The results of this study suggest that the OEV protein cargoes released under hormone regulation change considerably during the four stages of the estrous cycle in mice. The ER plays a crucial role in endocrine function and reproduction [28], and our results showed that the ER expression level in OEVs changes according to the stage of the natural estrous cycle in mice. The ER is not only present in the nucleus, but also on the plasma membrane [29]; however, the ER may also be detected in the released OEVs because extracellular vesicles are translocated into the cytoplasm via nuclear pores [30]. The physiological significance of the highest level of expression of the ER at the diestrus stage implies that the numbers and the types of the source cells of OEVs, the ciliated and secretory cells [21], may vary, as shown in Fig. 6. Moreover, the CD9 and Hsc70 levels in OEVs fluctuated across four stages, as previously reported [16]. CD9 and Hsc70 are also common exosomes biomarkers [16, 23, 31]. Besides, Hsc70 is also regarded not only as a biomarker but also as a positive control, as shown by Alminana [24]. The results of immunoelectron microscopy showed black gold particles (Figs. 3 and 4), indicating that ER and CD9 in the OEV were specifically recognized by the anti-ER and anti-CD9 antibodies, respectively, although the morphology of OEVs (Figs. 3 and 4) was different from that in Figs. 2 and 6. The OEV morphology is affected by the experiment conditions, including the chemical fixation and contrasting methods [33]. In previous studies using a bovine model, 170 among 336 protein contents of OEVs were differentially abundant across the estrous cycle [24], and several metabolites, including glucose-1-phosphate and methionine that regulate sucrose, glucose, and lactose metabolism, were significantly different across the estrous cycle [24]. Therefore, our results are consistent with the conclusion that the metabolites or contents of extracellular vesicles change during the different stages of the estrous cycle [24, 32].

Currently, variables determining the homogeneity and heterogeneity of the size distribution and composition of vesicles remain unclear, and it is speculated that vesicle size depends on the type of membrane phospholipids and the presence or absence of particular membrane proteins, and the composition may link with the biogenesis pathway and the cells of their origin [34]. However, our results strongly imply that the size distribution and the cargo of OEVs are associated with the physiological status of host cells, as well as influenced by multiple hormones that regulate the estrous cycle because the hormones may control the biogenesis pathway, the resource cells, and further the membrane proteins of OEVs. This result is consistent with a previous study, which showed that estrogen induces increased secretion of smaller-sized vesicles [35]. Additionally, the ciliated and secretory cells in the oviduct at different stages may be influenced by the following hormones in vivo that modulate the morphology of cells in the vaginal canal[18]: the levels of E2 [36] and PRL [37] sharply increase, and LH and FSH release into the circulation [38, 39] at the proestrus stage, which is similar to the human menstrual cycle [40]; the levels of FSH and PRL reach the peaks [36, 37] [41, 42], and E2 levels decline [36, 37] at the estrus stage; the levels of P4 and E2 start rising [36, 43, 44] at the metestrus stage; and the levels of P4 reach the peaks [37, 45, 46] at the diestrus stage. However, the detailed mechanism by which hormones act remains to be further elucidated.

In this study, we successfully observed the status of OEVs released by the oviduct at four stages in the estrous cycle in mice through ultrastructural observation with TEM. The results indicated that the OEV amount is higher at the proestrus and estrus stages and lower at the metestrus and diestrus stages. The observations of the oviduct ultrastructure in this study are consistent with the previously reported features of the murine oviduct [47], including the presence of cilia on the surface of the epithelial lining of ciliated and secretory cells; however, the previous study did not discuss the released extracellular vesicles. It has been reported that the OEVs arise from the apocrine pathway [5]. In this study, we showed the status of OEVs at four stages during the estrous cycle; however, the cilia could not be observed during the metestrus and diestrus stages. Nevertheless, this does not indicate the absence of cilia, because the images showed only a part of one or several cells and not the whole of the oviduct tissue. Based on our results, we propose that the cilia are less common at the metestrus and diestrus stages in mice than those at the proestrus and estrus stages. Thus, the results of this study are consistent with the numbers of the ciliated and secretory cells reported during the estrous cycle in the previous study [48]. Taken together, our study confirmed the release of OEVs from the oviduct and revealed the OEV status during the natural estrous cycle in mice.

In addition, our results suggest that the natural estrous cycle may be physiologically more beneficial for OEV investigation than the superovulated cycle in the report [5]. On one hand, the natural estrous cycle reflects the physiological status of mice, and the size distribution and the types and numbers of cargoes of the release OEV are not affected by the exogenous hormones, pregnant mare serum gonadotropin (PMSG), and human chorionic gonadotropin (HCG) that involved in superovulation. On the other hand, the isolation of OEVs in the natural estrous cycle saves the experimental time and is more convenient than that in the superovulated cycle, because it takes < 1 day to identify mice using the natural estrous cycle, compared to several days required to achieve superovulation. Admittedly, it is advantageous that the superovulation brings a population of females all into estrus unless special purpose at individual stages in estrus cycle is investigated.

This study clarifies the characteristics and dynamics of OEVs at different stages of the estrous cycle and provides new insights on how to obtain suitable OEVs or extracellular vesicles for potential clinical applications. The clinical application of OEVs may mainly provide the treatments of in vitro fertilization for the higher pregnancy rates in the scenarios of the assistant reproductive technology [11]. The clinical application of OEVs putting into practice still needs to be further developed although OEVs are advantageous to gamete fertilization and embryo development. There are many facing challenges or questions to be addressed: what is the standardization of isolation and purification methods for OEVs; how are the homogenous OEVs with different molecular cargoes obtained; what is the specific molecular cargo in OEVs acting on the recipient cell; what is the target molecule in the recipient cell by acted by OEVs; and what are the signaling networks and mechanisms by which OEVs acts on the recipient cells. Accordingly, this study may offer the clue that the different size distribution of OEVs with different cargoes could be produced and standardized via hormones control.

The limitation of this study has to be noted: the mechanisms by which the size of OEVs differ through the stages of the estrous cycle need to be further revealed in the future, especially which hormone(s) controls the size distribution and cargoes of the released OEVs; only protein cargoes and only three proteins of OEVs through different stages were investigated in the study; and more types and numbers of molecular cargoes of OEVs including proteins, mRNA, siRNA, and lipids need to be further explored in the future.

## Conclusions

In summary, the size distribution characteristics and the ER cargo of OEVs are different and vary across the proestrus, estrus, metestrus, and diestrus stages of the estrous cycle in mice. OEVs are released by the oviduct, which has a dynamic ultrastructure. The results of this study suggest that the size distribution, at least partial protein cargo, and status of released OEVs are influenced by the estrous cycle in mice, which may be associated with sex hormone levels in vivo, although the detailed mechanism requires further study. This study contributes to further understanding the characteristics and dynamics of OEVs at different stages of the estrous cycle; hence, it may be useful for revealing physiological and pathological changes associated with the estrous cycle. The study also provides clues to obtain OEVs or extracellular vesicles with uniform size and stable protein cargoes from other sources, which may have clinical applications.

## Data Availability

All data generated or analyzed during this study are included in this published article and available from the corresponding author.
